# Amplified Intraindividual Variability in Motor Performance in Stroke Survivors: Links to Cognitive and Clinical Outcomes

**DOI:** 10.1002/brb3.70365

**Published:** 2025-02-19

**Authors:** Stefan Delmas, Anjali Tiwari, Han‐Yun Tseng, Sharon N. Poisson, Manfred Diehl, Neha Lodha

**Affiliations:** ^1^ Department of Health and Exercise Science Colorado State University Fort Collins Colorado USA; ^2^ Department of Human Development and Family Studies Colorado State University Fort Collins Colorado USA; ^3^ Department of Neurology University of Colorado Aurora Colorado USA

**Keywords:** consistency, goal‐directed movements, cognitive function

## Abstract

**Background:**

Intraindividual variability (IIV) in motor performance reflects unintentional fluctuations in the motor output across repeated attempts. Behavioral variability in older adults has been linked to impaired neuronal integrity and cognitive decline. Despite this, the traditional motor assessments in stroke have neglected to characterize IIV in motor performance also known as “motor inconsistency.” Therefore, the aim of this study was to investigate the impact of stroke on motor inconsistency and its relationship with cognitive and clinical outcomes.

**Methods:**

Sixty‐six stroke survivors and 32 healthy older adults performed 30 trials of a goal‐directed task to match a force‐time target of 10 N in 180 ms. To measure motor inconsistency, we applied a well‐established approach to measuring IIV from the cognitive aging literature that accounts for the inherent, systematic effects of practice and mean‐level performance on IIV. In addition, participants completed domain‐specific cognitive evaluations and global clinical assessments. Domain‐specific cognitive evaluations assessed episodic memory, visuospatial processing, processing speed, and executive function. Global clinical assessments included years of education as a proxy of cognitive reserve, the Dementia Rating Scale‐2 (DRS‐2), ankle strength, and the Modified Rankin Score (mRS).

**Results:**

Stroke survivors exhibited greater motor inconsistency compared with healthy older adults. Declines in domain‐specific cognitive function, particularly executive dysfunction, predicted motor inconsistency in stroke survivors. Cognitive reserve and mRS emerged as significant predictors of motor inconsistency.

**Conclusions:**

Stroke significantly impairs the ability to perform a motor task with consistency. Compromised executive function following stroke is associated with increased motor inconsistency. Interestingly, reduced cognitive reserve and greater functional disability are linked to increased motor inconsistency in stroke survivors. These findings highlight that inconsistency is an important indicator of motor dysfunction following stroke that is linked to cognitive and clinical outcomes and may serve as an important target for stroke rehabilitation.

## Graphical abstract

Stroke survivors exhibit greater intraindividual variability (IIV) in motor performance. The elevated IIV in motor performance is linked to executive dysfunction, reduced cognitive reserve, and greater functional disability. These findings highlight motor inconsistency as a critical marker of post‐stroke motor dysfunction and a potential target for rehabilitation interventions.

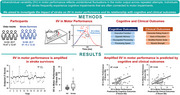

## 1 Introduction

Unintentional fluctuations in motor output across repeated attempts are a fundamental feature of human performance (Christou 2011; Yacoubi and Christou 2024). These fluctuations, reflecting within‐person inconsistency in motor behavior, are termed intraindividual variability (IIV) in motor performance, or “motor inconsistency.” Motor inconsistency increases with age and affects functional performance, leading to reduced movement accuracy (Haynes et al. 2017; Hultsch et al. 2000, 2002; Sofia Costa et al. 2019), inefficient task learning (Christou 2009), and difficulty in navigating environments (Lodha et al. 2016). Despite this, motor impairments in neurological diseases are conventionally measured using mean‐level performance and overlook the importance of consistent performance.

Stroke commonly leads to motor impairments, including reduced strength, speed, accuracy, and increased within‐trial variability (Kawahira et al. 2005; Lindberg et al. 2012; Lodha, Patel, Casamento‐Moran, Hays, et al. 2019; Mukherjee et al. 2013; Patel et al. 2020). However, the measurement of IIV following stroke is often neglected. Seminal findings in the cognitive aging literature show that IIV in behavior is a robust indicator of neural integrity (Burton et al. 2002, 2006; Fjell et al. 2011; Jackson et al. 2012; MacDonald et al. 2006; Stuss et al. 1994). This raises the possibility that neuronal damage from stroke may also increase fluctuations in motor output. However, systematic investigations on the impact of stroke on motor inconsistency are notably absent.

Cognitive processes are central to motor control. For example, executive function is crucial for planning and organizing the execution of a movement (Elliott 2003). Concurrent with motor dysfunctions, individuals with stroke frequently experience cognitive impairments (Carter et al. 2017; Hachinski et al. 2006; Hyndman et al. 2008; Liu et al. 2017; Nys et al. 2007; Teasell et al. 2015). These post‐stroke cognitive impairments negatively influence motor function. For instance, visuospatial impairment following stroke is associated with impaired manual dexterity (Sunderland et al. 1999). Similarly, compromised executive function is associated with poor gait performance (Hayes et al. 2013). Given that daily activities require motor‐cognitive integration, understanding how cognitive dysfunction relates to motor inconsistency post‐stroke is critical. Therefore, we aimed to examine the association between domain‐specific cognitive impairment and motor inconsistency in stroke survivors.

A robust experimental model to study motor inconsistency is the goal‐directed task (Carlton et al. 1993; Carlton and Newell 1988; Christou and Carlton 2001, 2002; Hu and Newell 2011; Kim et al. 1999). The goal‐directed task features fast, goal‐directed, pulse contractions (contractions accomplished in < 200 ms) completed over multiple trials (Carlton et al. 1993; Carlton and Newell 1988; Christou and Carlton 2001, 2002; Hu and Newell 2011; Kim et al. 1999). This task exemplifies cognitive contribution to motor performance for the following three reasons. First, the use of brief visual cues demands swift processing speed. Second, performance feedback after each trial requires visuospatial processing and episodic memory to improve in subsequent trials. Finally, the rapid nature of the task requires participants to preplan their movements, emphasizing executive function (Casamento‐Moran et al. 2017; Delmas et al. 2021; Desmurget and Grafton 2000; Ghez and Gordon 1987; Seidler et al. 2004; Shadmehr et al. 2010). Together, processing speed, visuospatial processing, episodic memory, and executive function, are integral for optimal performance in the goal‐directed task.

This study aimed to address two primary questions. First, *what is the impact of stroke on motor inconsistency?* We hypothesize that behavioral fluctuations attributed to neural integrity will extend to the motor domain such that stroke survivors will exhibit greater motor inconsistency than healthy older adults. Second, *what is the predictive relevance of domain‐specific cognitive outcomes on motor inconsistency?* Given their importance in the goal‐directed task, we hypothesized that processing speed, visuospatial processing, episodic memory, and executive function would significantly predict motor inconsistency in stroke survivors.

Clinicians rely on broad motor and cognitive assessment tools to characterize disability and recovery after stroke (Ada et al. 2006; Canning et al. 2004; Faria‐Fortini et al. 2011, 2017; Harrison et al. 2013; Rosenich et al. 2020). Exploring the relationship between clinical outcomes and fundamental mechanistic variables like motor inconsistency will enhance our understanding of the biological mechanisms behind disease severity and help identify targets for assessing treatment effectiveness. Therefore, we propose a secondary question: *What is the predictive relevance of clinical outcomes on motor inconsistency?* We hypothesized that global clinical assessments of cognitive and motor function would significantly predict motor inconsistency in stroke survivors.

## 2 Methods

### 2.1 Participants

Sixty‐six stroke survivors and 32 healthy older adults volunteered to participate in the study. Detailed participant demographics and characteristics are presented in Table 1. Inclusion criteria for the stroke participants included: (1) > 21 years of age at the time of study participation, (2) diagnosis of stroke (ischemic or hemorrhagic) at least 9 months before enrollment, and (3) a minimum of 10° of active movement in the paretic ankle (combined dorsiflexion and plantar flexion). Exclusion criteria for all participants included < 21 years of age, self‐reported presence of any neurological disorder other than stroke (e.g., traumatic brain injury, Parkinson's disease, epilepsy), substance abuse, aphasia, untreated sleep or anxiety disorders, or uncorrected vision and hearing impairments. The Institutional Review Board (IRB) of Colorado State University (CSU) approved the experimental procedures.

**TABLE 1:** Descriptive statistics (*N* = 98).

Variable	Stroke (*n* = 66)	Healthy older (*n* = 32)	*p*
Sociodemographic characteristics			
Age, years, mean (SD)	67.15 (12.32)	70.67 (12.67)	0.191
Women, *n*	28	20	0.115
Ethnicity, *n*			
Non‐Hispanic White	61	27	
Asian	2	3	
African American	2	0	
Hispanic	0	2	
Other	1	0	
Type of stroke, *n*			
Ischemic	52	—	
Hemorrhagic	10	—	
Both	1	—	
Unknown	3	—	
Years since stroke, mean (SD)	3.91 (5.56)	—	
Side of the lesion, *n*			
Right	21	—	
Left	28	—	
Both	5	—	
Unknown	12	—	
Lesion location, *n*			
Infratentorial	8	—	
Supratentorial	41	—	
Both	5	—	
Unknown	12	—	

Abbreviation: SD, standard deviation.

### 2.2 Experimental Protocol

The experimental protocol consisted of a single baseline session lasting approximately 90 min during which participants performed an isometric ankle goal‐directed task, a battery of domain‐specific cognitive tests, and clinical assessments of global cognitive and motor health.

### 2.3 Goal‐Directed Task

**FIGURE 1:**
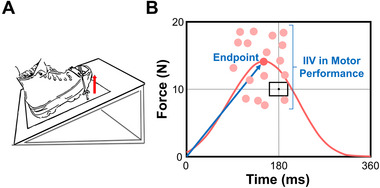
Goal‐directed task. (A) Schematic representation of the set‐up showing the position of the foot. (B) Representative goal‐directed performance. Participants were required to match a force target of 10 N in 180 ms. The relative distance from the origin to peak of the bell‐shaped performance represents the endpoint. Intraindividual variability (IIV) in motor performance represents the trial‐to‐trial inconsistency in endpoint.

Participants sat upright facing a 32‐in. monitor (Samsung DC32E, Samsung Electronics America, resolution: 1920 × 1080, refresh rate: 50 Hz), positioned 1.85 m away at eye level. The hip joint was flexed to 90°, the knee was flexed to 90°, and the paretic ankle was in a neutral position. The foot rested on a customized foot device that we secured by straps over the metatarsals to ensure an isolated dorsiflexion of the ankle (Figure 1A). The force exerted during the goal‐directed contractions was measured using a force transducer (Model 41, Honeywell, Columbus, OH, USA) located parallel to the force direction on the customized foot device. The ankle force signal was sampled at 1000 Hz using a NI‐DAQ card (Model USB‐6343 (BNC), National Instruments) and stored on a personal computer for offline analysis. The monitor displayed the contraction produced by ankle dorsiflexion using a custom‐written program in Matlab (Math Works Inc., Natick, MA, USA). All participants confirmed that they could see the screen clearly.

We adapted the goal‐directed protocol from previously published studies (Casamento‐Moran et al. 2015; Christou et al. 2007; Delmas et al. 2021; Lodha et al. 2019). Participants performed 30 goal‐directed contractions. To perform the task correctly, participants had to produce a target force of 10 N in 180 ms, creating a bell‐shaped curve with a clear peak using ankle dorsiflexion. The task consisted of three phases: GET READY, MOVE, and FEEDBACK. In the GET READY phase, a red target appeared on the monitor for 2 s, signaling participants to prepare. During the MOVE phase, the target turned green for 3 s, prompting participants to perform the goal‐directed contractions. Participants had 3 s to begin, with force‐time recording starting at the onset of contraction. No online feedback was provided during the task to avoid self‐corrections. In the FEEDBACK phase, participants received visual feedback on their performance relative to the target force and time for 5 s.

#### 2.3.1 IIV in Motor Performance (Motor Inconsistency)

We examined motor inconsistency by quantifying the trial‐to‐trial variability in the endpoint during the goal‐directed task (Figure 1B). The endpoint was calculated from peak force and time‐to‐peak force. After converting these values to comparable units (%; Equations 1 and 2), the endpoint was derived as the hypotenuse between them (Equation 3).

(1) $$\mathrm{Peak}\;\mathrm{force}\;\left(\%\right)=\frac{\mathrm{Peak}\;\mathrm{force}\;\left(N\right)}{\mathrm{Target}\;\mathrm{peak}\;\mathrm{force}\;\left(N\right)}\times 100$$

(2) $$\begin{array}{ccc} & & \mathrm{Time}-\mathrm{to}-\mathrm{peak}\;\mathrm{force}\;\left(\%\right)=\\ & & \frac{\mathrm{Time}-\mathrm{to}-\mathrm{peak}\;\mathrm{force}\;\left(\mathrm{ms}\right)}{\mathrm{Target}\;\mathrm{time}-\mathrm{to}-\mathrm{peak}\;\mathrm{force}\;\left(\mathrm{ms}\right)}\times 100 \end{array}$$

(3) $$\begin{array}{ccc} & & \mathrm{Endpoint}\;\left(\%\right)=\\ & & \sqrt{{\left(\mathrm{Peak}\;\mathrm{force}\;\left(\%\right)\right)}^{2}+{\left(\mathrm{time}-\mathrm{to}-\mathrm{peak}\;\mathrm{force}\;\left(\%\right)\right)}^{2}}\; \end{array}$$

Data outside the mean ± 3 SD range were considered outliers. No values fell below the mean − 3 SD. Approximately 1.95% of data for healthy adults and 1.78% for stroke survivors exceeded the mean + 3 SD and were imputed by replacing them with the mean + 3 SD.

To compute motor inconsistency, we applied the established statistical approach developed by Hultsch et al. (2000). We chose this approach because it provides a conservative measure of IIV by controlling for the systematic effects of practice and group means (Burton et al. 2002; Hultsch et al. 2000, 2002; Hultsch and MacDonald 2004; Sofia Costa et al. 2019). To eliminate these effects, we entered trial and group means as predictors of endpoint in a linear regression model and computed the resultant standardized endpoint residuals (*z* scores). Next, we converted the resulting *z* scores for each trial into standardized T‐scores and computed motor inconsistency as the standard deviation of endpoint T‐scores across 30 trials.

### 2.4 Domain‐Specific Cognitive Assessments

We determined that episodic memory, visuospatial processing, processing speed, and executive function are essential for performing the goal‐directed task. Below, we describe how we assessed these domain‐specific cognitive functions and how they are relevant to the goal‐directed task.

#### 2.4.1 Episodic Memory

We assessed episodic memory using the Hopkins Verbal Learning Test (HVLT) (Belkonen 2018). We asked participants to recall a list of words over several trials and recorded the total number of words recalled. Lower scores in HVLT reflect poorer episodic memory. Episodic memory refers to the ability to form and retrieve memories of past events (Danieli et al. 2023). Before each trial in the goal‐directed task, we provide visual feedback on their past performance. Episodic memory is needed to recall their past performance to improve on the subsequent trial.

#### 2.4.2 Visuospatial Processing

We assessed visuospatial processing using the Digit Symbol Substitution Test (DSST) (Jaeger 2018). Participants matched symbols to corresponding numbers as quickly as possible within a set time limit. Lower scores on DSST demonstrate poorer visuospatial processing. Visuospatial processing involves skills such as processing changes in position and accurately locating objects in space (Stiles et al. 2020). During the goal‐directed task, visuospatial processing is essential for locating the target on the screen and translating spatial information into motor action.

#### 2.4.3 Processing Speed

We assessed processing speed using Part A of the Trail Making Test (TMT‐A) (Bowie and Harvey 2006). We recorded the time taken for participants to draw lines sequentially connecting encircled numbers. Longer times on the TMT‐A are indicative of slower processing speed. Processing speed is the ability to perceive, interpret, and respond to stimuli. Fast processing is required to respond promptly to limited‐time visual cues in the goal‐directed task.

#### 2.4.4 Executive Function

We assessed executive function using Part B of the Trail Making Test (TMT‐B) (Reitan 1992). We recorded the time taken for participants to draw lines alternating between numbers and letters (e.g., 1‐A‐2‐B). Longer times on the TMT‐B are indicative of poorer executive function. Executive function encompasses a variety of high‐level cognitive abilities such as inhibitory control, attention shifting, working memory, and planning (Barkley 1997; Best and Miller 2010; Chung et al. 2014; Diamond 2013; Miyake et al. 2000; Weyandt 2009; Zelazo and Müller 2010). Given the rapid nature of the contractions and the absence of online feedback during the goal‐directed task, participants must pre‐plan their contraction to hit the target (Casamento‐Moran et al. 2017; Desmurget and Grafton 2000; Gordon and Ghez 1987; Seidler et al. 2004; Shadmehr et al. 2010).

### 2.5 Global Assessments of Cognitive and Motor Function

#### 2.5.1 Global Assessments of Cognitive Function

We globally assessed cognitive function using the Dementia Rating Scale‐2 (DRS‐2) and years of education. The DRS‐2 provides a comprehensive assessment of overall cognitive severity with higher scores indicating better cognition (Lopez et al. 2021). In addition, the DRS‐2 is an updated form of the DRS that includes age‐ and education‐adjusted scoring (Jurica et al. 2001). Cognitive reserve is conceptualized as a resilience‐related feature of brain function that mitigates the impact of brain pathology on clinical outcomes (Rosenich et al. 2020). Years of education represent a robust measure of cognitive reserve whereby more years of education indicate greater cognitive reserve (Rosenich et al. 2020).

#### 2.5.2 Global Assessments of Motor Function

We globally assessed motor functions using the Modified Rankin Scale (mRS) and ankle motor strength. The mRS is a widely used clinical tool for measuring the degree of disability among stroke survivors where higher scores indicate greater disability (Ada et al. 2006; Canning et al. 2004; Faria‐Fortini et al. 2011, 2017; Harrison et al. 2013). In addition, strength is an important clinical indicator of progress toward stroke motor recovery (Boissy et al. 1999; Stock et al. 2019). We measured ankle motor strength using an electronic manual muscle tester (Lafayette Manual Muscle tester, 01165, IEC 60601‐1‐2; 2007‐03). While participants lay on a bed flat on their backs, we placed the muscle tester against their metatarsals and instructed them to maximally dorsiflex their feet without bending their knees. We selected the highest value recorded from two trials in the left and right ankle. Next, we computed relative strength as the paretic/non‐paretic × 100 (non‐dominant/dominant × 100 for healthy older adults). Therefore, values below 100% indicate that the strength of the paretic ankle is lower than the strength of the non‐paretic ankle.

### 2.6 Statistical Analysis

We used independent *t*‐tests for continuous variables and chi‐square tests for categorical variables to compare demographic, domain‐specific cognitive, and global assessment outcomes between stroke survivors and healthy older adults. To assess stroke's impact on motor inconsistency and examine sex as a biological factor, we conducted a 2 Group × 2 Sex analysis of variance. We use Cohen's *d* to report the effect size of group differences. To determine the predictive relevance of domain‐specific cognitive outcomes on motor inconsistency in stroke survivors, we conducted a multiple linear regression analysis with episodic memory, visuospatial processing, processing speed, and executive function scores as predictor variables and motor inconsistency as the criterion variable. Similarly, to determine the predictive relevance of global clinical outcomes on motor inconsistency, we conducted a multiple linear regression analysis with years of education, DRS‐2, ankle strength, and mRS scores as predictor variables and motor inconsistency as the criterion variable. All statistical significance tests were conducted with the alpha level set at 0.05 using SPSS 27.0 (IBM, Armonk, NY, USA) corrected for multiple comparisons using FDR correction (Benjamini and Hochberg 1995).

To achieve 80% power at an alpha level of 0.05 and a moderate effect size, 30 participants per group were needed to address the first primary question. To ensure a robust n/k ratio (data points/number of predictors) ≥ 15:1, a minimum sample size of 60 participants was needed to address the second primary question.

## 3 Results

### 3.1 Participant Demographics

The stroke and healthy older adult groups did not differ in age or sex (Table 1). The primary ethnicity in both groups was White/Caucasian (> 84%). The stroke group contained two participants with Asian ethnicity and two participants with African American ethnicity. The healthy older adults group had three participants with Asian ethnicity, and two participants with Hispanic ethnicity.

### 3.2 Motor Inconsistency

**FIGURE 2:**
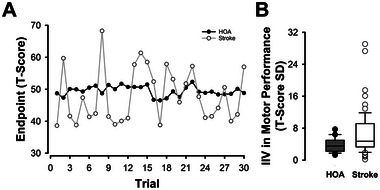
Intraindividual variability (IIV) in motor performance between stroke survivors and healthy older adults (HOA). (A) Representative endpoint residual T‐scores (purified for group and trial) across the 30 trials for a stroke and healthy older adult participant. IIV in motor performance is represented by the greater variation in endpoint across trials for the stroke participants relative to the healthy older adult participant. (B) At the cohort level, stroke survivors exhibited significantly greater IIV in motor performance than healthy older adults.

Group means and SDs on motor inconsistency are presented in Table 2. A Group × Sex ANOVA on motor inconsistency revealed a significant main effect of group on motor inconsistency, *F*(1,96) = 8.80, *p* = 0.004, *η* ^2^ = 0.09 (Figure 2). However, we found no main effect of sex, *F*(1,96) = 0.21, *p* = 0.65, *η* ^2^ < 0.01, on motor inconsistency. In addition, the group by sex interaction on motor inconsistency was not significant, *F*(1,96) = 0.55, *p* = 0.46, *η* ^2^ < 0.01.

**TABLE 2:** Intraindividual variability (IIV) in motor performance, domain‐specific cognitive functioning, and global assessment outcomes for stroke survivors and healthy older adults.

	Stroke (*n* = 66)	Healthy older (*n* = 32)	*t*	*p*	Cohen's *d*
Goal‐directed task					
IIV in motor performance (T‐score SD)	6.61 (5.39)	3.74 (1.78)	−3.91	< 0.001	0.63
Domain‐specific cognitive functioning					
Episodic memory (# of words)	5.67 (1.94)	9.59 (1.85)	9.54	< 0.001	2.06
Visuospatial processing (# completed)	47.82 (18.11)	61.75 (12.97)	3.89	< 0.001	0.84
Processing speed (s)	51.80 (52.24)	31.79 (9.87)	−3.00	0.004	0.46
Executive function (s)	118.56 (77.52)	79.14 (40.58)	−3.30	0.001	0.58
Global assessments					
Years of education	15.88 (2.59)	16.81 (2.60)	1.67	0.147	0.36
DRS‐2 (score)	8.29 (3.11)	10.91 (1.69)	5.39	< 0.001	0.96
mRS (score)	1.42 (0.93)	—			
Ankle strength (%)	0.94 (0.22)	1.00 (0.16)	1.26	0.210	0.27

*Note*: Values are reported as mean (SD).

Abbreviations: DRS‐2: Dementia Rating Scale‐2; mRS: Modified Ranking Scale.

### 3.3 Domain‐Specific and Global Assessments of Cognitive and Motor Function

Table 2 presents the domain‐specific and global assessments of cognitive and motor function for stroke survivors and healthy older adults. Results show that stroke survivors performed worse in episodic memory, visuospatial processing, processing speed, and executive function compared to the healthy group. In addition, the stroke group had lower DRS‐2 scores, indicating poorer global cognitive functioning. However, no significant differences were found between the groups in cognitive reserve or ankle motor strength.

### 3.4 Predictive Relevance of Domain‐Specific Cognition for Motor Inconsistency in Stroke Survivors

**FIGURE 3:**
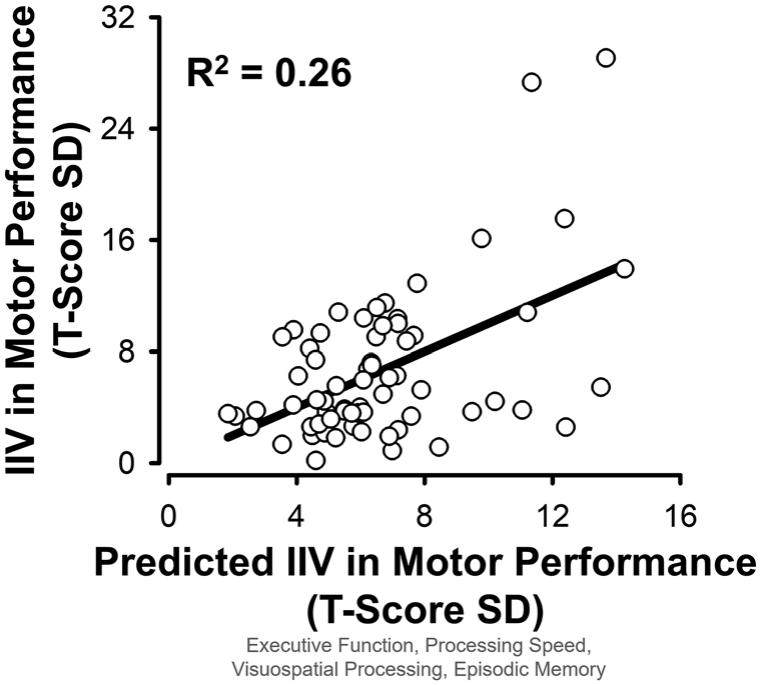
Multiple linear regression model predicts intraindividual variability (IIV) in motor performance from domain‐specific cognition. Deterioration in executive function, processing speed, visuospatial processing, and episodic memory predicted IIV in motor performance in stroke survivors.

All domain‐specific cognitive measures together accounted for a significant amount of variance in motor inconsistency, *F*(4,61) = 5.47, *R* ^2^ = 0.26, *p* = 0.001 (Figure 3). An *R* ^2^ of 0.26 qualifies as a large effect size (Cohen 2013). Notably, executive function emerged as the only significant predictor of motor inconsistency (Table 3, Model 1).

**TABLE 3:** Regression coefficients of domain‐specific cognitive functioning and global assessments for intraindividual variability (IIV) in motor performance in stroke survivors.

	*b*	SE *b*	*β*	*t*	*p*
Model 1: Domain‐specific cognitive functioning and IIV in motor performance					
Episodic memory	−0.628	0.34	−0.23	−1.86	0.068
Visuospatial processing	−0.028	0.05	−0.09	−0.53	0.596
Processing speed	< 0.001	0.00	−0.19	−1.12	0.268
Executive function	< 0.001	0.00	0.46	2.58	**0.012**
Model 2: Global assessments and IIV in motor performance					
Years of education	−0.605	0.24	−0.29	−2.56	**0.013**
DRS‐2	−0.276	0.20	−0.16	−1.37	0.176
mRS	1.947	0.74	0.34	2.64	0.010
Ankle strength	1.384	3.00	0.06	0.46	**0.646**

### 3.5 Predictive Relevance of Global Assessments of Cognitive and Motor Function for Motor Inconsistency in Stroke Survivors

**FIGURE 4:**
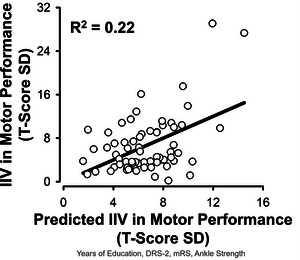
Multiple linear regression predicts intraindividual variability (IIV) in motor performance from global assessments. The years of education, Dementia Rating Scale‐2 (DRS‐2), Modified Rankin Scale (mRS), and ankle strength predicted IIV in motor performance in stroke survivors.

All global assessment outcomes together accounted for a significant amount of variance in motor inconsistency, *F*(4,61) = 4.33, *R* ^2^ = 0.22, *p* = 0.004 (Figure 4). An *R* ^2^ of 0.22 qualifies as a medium effect size (0.13 ≤ *R* ^2^ < 0.26) (Cohen 2013). Specifically, years of education and mRS emerged as significant predictors of motor inconsistency (Table 3, Model 2).

## 4 Discussion

This study addressed three questions. First, we determined the impact of stroke on IIV in motor performance (motor inconsistency). Our findings indicate that individuals with stroke demonstrate significantly higher motor inconsistency than healthy older adults. Second, we determined if domain‐specific cognition significantly predicts motor inconsistency in stroke survivors. Lower executive function emerged as a key predictor of greater motor inconsistency. Finally, we determined if clinical outcomes significantly predict motor inconsistency in stroke survivors. We found that motor inconsistency is predicted by clinically meaningful motor and cognitive outcomes with an emphasis on mRS and years of education. This study provides the first evidence that motor inconsistency is a significant marker of motor dysfunction in stroke survivors, that is influenced by compromised executive function and is linked to clinical outcomes.

### 4.1 Motor Inconsistency Is Exacerbated in Individuals Following Stroke

Goal‐directed movements such as catching a falling object or adjusting posture to avoid tripping and falling are common. Most investigations on motor impairments in stroke survivors to date focus on quantifying mean‐level performance (Eng et al. 2002; Rohrer et al. 2002). Few stroke studies on motor output variability have examined within‐trial variations, while between‐trial variability has been largely overlooked (Chang et al. 2013; Chow and Stokic 2011; Cirstea and Levin 2000; Lodha et al. 2012, 2010; Patel and Lodha 2019). Although within‐ and between‐trial variability fall under the umbrella term of motor output variability, they are theoretically unrelated to each other and are potentially linked with distinct biological mechanisms (Yacoubi and Christou 2024).

Prior research on motor inconsistency is mainly centered on healthy older adults, showcasing that they exhibit greater motor inconsistency while performing goal‐directed contractions with the finger (Christou et al. 2007) and leg (Christou and Carlton 2001)^.^ Using rigorous methodology, we found that stroke greatly impairs the ability to produce consistent motor output. These findings point to the possibility that IIV may reflect an intrinsic influence of stroke‐related deterioration in neuronal integrity on motor performance. To our knowledge, our study provides the first evidence that inconsistency is a prominent feature of poststroke motor impairment.

The physiological mechanisms underpinning IIV in motor performance are currently not well understood. IIV likely reflects disruptions in neural mechanisms responsible for stable and consistent performance (Burton et al. 2002, 2006; Fjell et al. 2011; Jackson et al. 2012; MacDonald et al. 2006; Stuss et al. 1994). For example, stroke‐related damage to key brain regions, including the prefrontal cortex and motor pathways, may lead to inefficient neural signal transmission, increased noise in motor output, and disrupted neural integrity resulting in amplified motor inconsistency. Although elevated IIV in motor performance can be subtle, it can significantly impact activities requiring precision and coordination and has been linked to disease severity (Yacoubi and Christou 2024). Rehabilitation interventions focusing on training stroke survivors to execute consistent movement patterns across various stages of recovery can potentially harness neural plasticity through a complex interaction of functional and structural brain changes (Lövdén et al. 2010). Reduced IIV in motor performance may lead to more efficient task processing and enhanced everyday functioning (Brehmer et al. 2014). Therefore, our findings emphasize that the impact of stroke on IIV in motor performance should be considered as an important indicator in stroke rehabilitation.

A key strength of this study is the robust methodology used to quantify motor inconsistency. Rather than relying on traditional measures like standard deviation, coefficient of variation, or approximate entropy, we implemented a rigorous approach established by Hultsch et al. in cognitive aging research (Burton et al. 2002; Hultsch et al. 2000, 2002; Hultsch and MacDonald 2004). This methodology offers two notable advantages. First, this approach provides a “purified” measure of motor inconsistency by accounting for systematic within‐person factors that would otherwise confound IIV (Burton et al. 2002; Hultsch et al. 2000, 2002; Hultsch and MacDonald 2004; Sofia Costa et al. 2019). Specifically, we controlled for practice effects and group differences in the mean level of motor performance which would likely inflate motor inconsistency. Importantly, our findings suggest that between‐group differences in motor inconsistency are not linked to age, sex, or muscle strength. The second advantage is that IIV was computed as residual T‐scores, an approach that facilitates easy comparison across different experimental tasks, conditions, or occasions. Therefore, this method offers a robust and standardized approach for quantifying motor inconsistency, enhancing the reliability and comparability of findings across diverse populations and experimental contexts.

### 4.2 Executive Function Is a Key Predictor of Motor Inconsistency in Individuals Following Stroke

Interestingly, we found that lower executive function is a key predictor of increased motor inconsistency in stroke survivors. This finding deserves attention for two reasons. First, the interaction between cognitive and motor processes is of critical importance to everyday function. Executive functions, such as planning motor actions and shifting attention (Barkley 1997; Best and Miller 2010; Chung et al. 2014; Diamond 2013; Miyake et al. 2000; Weyandt 2009; Zelazo and Müller 2010), enable individuals to integrate sensory information from multiple sources and are fundamental for successfully achieving a movement goal (Elliott 2003). Lower executive function has been linked to impaired mobility in stroke survivors, such as slower performance during dual‐task walking (Hayes et al. 2013) and lower scores on balance assessments (Hyndman et al. 2008; Hyndman and Ashburn 2003; Liu‐Ambrose et al. 2007; Stapleton et al. 2001). By highlighting the connection between executive function and motor inconsistency, our study advances the understanding of how post‐stroke cognitive impairments influence motor inconsistency.

Second, the emphasis on compromised executive function on motor inconsistency supports the idea that motor inconsistency potentially reflects trial‐to‐trial variations in motor planning. The experimental model we used to quantify motor inconsistency involved rapid (< 200 ms) open‐loop contractions completed over multiple trials. Such contractions are primarily driven by preplanned descending cortical commands and are not influenced by online feedback (Casamento‐Moran et al. 2017; Desmurget and Grafton 2000; Gordon and Ghez 1987; Seidler et al. 2004; Shadmehr et al. 2010). Variations in cognitive processes that contribute to the development and structuring of the motor plan may lead to increased inconsistency in motor performance (Gordon et al. 1994). Indirect evidence from aging studies also shows that motor inconsistency is associated with altered coordination patterns between agonist‐antagonist muscles (Christou 2011). Therefore, we propose that motor inconsistency may serve as a valuable indicator for disrupted motor planning in individuals with stroke.

### 4.3 Global Motor and Cognitive Assessments Predict Motor Inconsistency in Stroke

A clinically significant finding is that motor inconsistency was predicted by global assessments of motor and cognitive performance that are routinely used for evaluating the degree of disability following stroke. Specifically, greater motor inconsistency was predicted by fewer years of education and higher mRS. Higher education has been shown to preserve cognition and delay poststroke dementia (Booth et al. 2013; Ojala‐Oksala et al. 2012; Rosenich et al. 2020), while mRS is the standard for evaluating stroke‐related disability (Ada et al. 2006; Canning et al. 2004; Faria‐Fortini et al. 2011, 2017; Harrison et al. 2013). Taken together, the association between motor inconsistency and clinical outcomes of cognitive and motor function suggests that motor inconsistency may be an informative behavioral outcome in clinical practice and could be considered in post‐stroke assessment protocols.

### 4.4 Clinical Implications

Current stroke rehabilitation typically targets improvements in mean‐level motor performance, often overlooking the importance of consistency (Duncan et al. 2011; Hornby et al. 2019). We argue that consistent movement is essential for motor learning and recovery after stroke. Increased performance variability can impede motor learning and recovery. Heightened motor inconsistency also undermines confidence in movement (Yacoubi and Christou 2024) and can impair the ability to navigate the environment (Lodha et al. 2016). Research in healthy older adults shows that motor training to reduce trial‐to‐trial variability leads to faster (Shmuelof et al. 2012) and more accurate (Hammerbeck et al. 2017) movements post‐training. Whether such training would be useful in stroke rehabilitation warrants further investigation. Our study provides preliminary evidence that motor inconsistency is amplified following stroke and is associated with compromised executive function and greater mRS. Therefore, we propose that consistency in motor performance should be an important goal of stroke motor rehabilitation.

### 4.5 Limitations and Further Considerations

Despite the strength of the study findings, there are several limitations that need to be acknowledged. First, this study does not address the effects of lesion location, size, or time since stroke on motor inconsistency. Second, amplified motor inconsistency may disrupt sensorimotor map formation, essential for learning new motor skills, potentially complicating stroke recovery (van Beers 2009; Van Vugt and Ostry 2018; Yacoubi and Christou 2024). However, its direct impact on skill acquisition remains unstudied. Third, the concepts of cognitive reserve and brain reserve are often used when discussing brain pathology and clinical manifestations. While our findings provide some insights into the relationship between IIV in motor performance and cognitive reserve, brain reserve is not considered. Future studies should consider the possible implication of cognitive reserve with brain reserve and how they impact IIV in motor performance in stroke survivors especially as it relates to various types of rehabilitation protocols. Fourth, our findings highlight motor inconsistency in the lower limbs. If IIV reflects central nervous system integrity, upper limb motor inconsistency may also be elevated, warranting further study. Fifth, another important limitation is that this study did not account for premorbid physical activity or history of rehabilitation. It is possible that differences in physical activity or changes due to rehabilitation before study procedures may interact with IIV in motor performance. Sixth, we did not account for the potential presence of comorbidities (e.g., diabetes, hypertension), which may play a role in the findings. Lastly, future studies should explore a more comprehensive battery of cognitive tests to thoroughly examine how cognition links to IIV in motor performance in stroke survivors.

## 5 Conclusion

In conclusion, this study demonstrates that stroke impairs the ability to execute consistent motor output. Inconsistency in motor performance is linked to compromised executive function and greater functional disability. These findings underscore the importance of quantifying IIV in motor performance in stroke survivors that may guide the development of effective rehabilitation interventions to improve motor recovery after stroke.

## Author Contributions

**Stefan Delmas**: writing–original draft, writing–review and editing, investigation, formal analysis. **Anjali Tiwari**: investigation, data curation. **Han‐Yun Tseng**: writing–original draft, writing–review and editing, formal analysis. **Sharon N. Poisson**: writing–review and editing. **Manfred Diehl**: writing–review and editing. **Neha Lodha**: conceptualization, methodology, resources, software, supervision, project administration, validation, visualization, funding acquisition, writing–review and editing.

## Ethics Statement

Research reported in this manuscript received ethical approval from the Institutional Review Board at Colorado State University.

## Consent

All participants provided informed consent to participate and were aware their data would be used to prepare publications.

## Conflicts of Interest

The authors declare no conflicts of interest.

### Peer Review

The peer review history for this article is available at https://publons.com/publon/10.1002/brb3.70365.

## Data Availability

The data generated and analyzed in this study are available from the corresponding author upon reasonable request.

## References

[brb370365-bib-0001] Ada, D. L. , N. O'Dwyer , and E. O'Neill . 2006. “Relation Between Spasticity, Weakness and Contracture of the Elbow Flexors and Upper Limb Activity After Stroke: An Observational Study.” Disability and Rehabilitation 28, no. 13–14: 891–897. 10.1080/09638280500535165. 16777777

[brb370365-bib-0002] Barkley, R. A. 1997. “Behavioral Inhibition, Sustained Attention, and Executive Functions: Constructing a Unifying Theory of ADHD.” Psychological Bulletin 121, no. 1: 65–94. 10.1037/0033-2909.121.1.65. 9000892

[brb370365-bib-0003] Belkonen, S. 2018. “Hopkins Verbal Learning Test.” In Encyclopedia of Clinical Neuropsychology, edited by J. S. Kreutzer , J. DeLuca , and B. Caplan , 1733–1735. Springer. 10.1007/978-3-319-57111-9_1127.

[brb370365-bib-0004] Benjamini, Y. , and Y. Hochberg . 1995. “Controlling the False Discovery Rate: A Practical and Powerful Approach to Multiple Testing.” Journal of the Royal Statistical Society: Series B (Methodological) 57, no. 1: 289–300.

[brb370365-bib-0005] Best, J. R. , and P. H. Miller . 2010. “A Developmental Perspective on Executive Function.” Child Development 81, no. 6: 1641–1660. 10.1111/j.1467-8624.2010.01499.x. 21077853 PMC3058827

[brb370365-bib-0006] Boissy, P. , D. Bourbonnais , M. M. Carlotti , D. Gravel , and B. A. Arsenault . 1999. “Maximal Grip Force in Chronic Stroke Subjects and Its Relationship to Global Upper Extremity Function.” Clinical Rehabilitation 13, no. 4: 354–362. 10.1191/026921599676433080. 10460123

[brb370365-bib-0007] Booth, A. J. , J. D. Rodgers , C. E. Schwartz , et al. 2013. “Active Cognitive Reserve Influences the Regional Atrophy to Cognition Link in Multiple Sclerosis.” Journal of the International Neuropsychological Society 19, no. 10: 1128–1133. 10.1017/S1355617713001082. 24050681

[brb370365-bib-0008] Bowie, C. R. , and P. D. Harvey . 2006. “Administration and Interpretation of the Trail Making Test.” Nature Protocols 1, no. 5: 2277–2281. 10.1038/nprot.2006.390. 17406468

[brb370365-bib-0009] Brehmer, Y. , G. Kalpouzos , E. Wenger , and M. Lövdén . 2014. “Plasticity of Brain and Cognition in Older Adults.” Psychological Research 78, no. 6: 790–802. 10.1007/S00426-014-0587-Z. 25261907

[brb370365-bib-0010] Burton, C. L. , D. F. Hultsch , E. Strauss , and M. A. Hunter . 2002. “Intraindividual Variability in Physical and Emotional Functioning: Comparison of Adults With Traumatic Brain Injuries and Healthy Adults.” Clinical Neuropsychologist 16, no. 3: 264–279. 10.1076/CLIN.16.3.264.13854. 12607140

[brb370365-bib-0011] Burton, C. L. , E. Strauss , D. F. Hultsch , A. Moll , and M. A. Hunter . 2006. “Intraindividual Variability as a Marker of Neurological Dysfunction: A Comparison of Alzheimer's Disease and Parkinson's Disease.” Journal of Clinical and Experimental Neuropsychology 28, no. 1: 67–83. 10.1080/13803390490918318. 16448976

[brb370365-bib-0012] Canning, C. G. , L. Ada , R. Adams , and N. J. O'Dwyer . 2004. “Loss of Strength Contributes More to Physical Disability After Stroke Than Loss of Dexterity.” Clinical Rehabilitation 18, no. 3: 300–308. 10.1191/0269215504CR715OA. 15137561

[brb370365-bib-0013] Carlton, L. G. , Y.‐T. Liu , and K. M. Newell . 1993. “Variability in Force Output With Variations in Characteristics of Force Production.” Journal of Biomechanics 26, no. 3: 329. 10.1016/0021-9290(93)90499-5.

[brb370365-bib-0014] Carlton, L. G. , and K. M. Newell . 1988. “Force Variability and Movement Accuracy in Space‐Time.” Journal of Experimental Psychology: Human Perception and Performance 14, no. 1: 24–36. 10.1037/0096-1523.14.1.24. 2964505

[brb370365-bib-0015] Carter, A. R. , M. P. McAvoy , J. S. Siegel , et al. 2017. “Differential White Matter Involvement Associated With Distinct Visuospatial Deficits After Right Hemisphere Stroke.” Cortex 88: 81–97. 10.1016/J.CORTEX.2016.12.009. 28081452 PMC5462627

[brb370365-bib-0016] Casamento‐Moran, A. , Y.‐T. Chen , M. Kwon , et al. 2015. “Force Dysmetria in Spinocerebellar Ataxia 6 Correlates With Functional Capacity.” Frontiers in Human Neuroscience 9: 184. 10.3389/fnhum.2015.00184. 25904859 PMC4389656

[brb370365-bib-0017] Casamento‐Moran, A. , Y.‐T. Chen , N. Lodha , B. Yacoubi , and E. A. Christou . 2017. “Motor Plan Differs for Young and Older Adults During Similar Movements.” Journal of Neurophysiology 117, no. 4: jn.00640.2016. 10.1152/jn.00640.2016. PMC5376608 28077666

[brb370365-bib-0018] Chang, S. H. , G. E. Francisco , P. Zhou , W. Z. Rymer , and S. Li . 2013. “Spasticity, Weakness, Force Variability, and Sustained Spontaneous Motor Unit Discharges of Resting Spastic–Paretic Biceps Brachii Muscles in Chronic Stroke.” Muscle & Nerve 48, no. 1: 85–92. 10.1002/MUS.23699. 23605647 PMC3691331

[brb370365-bib-0019] Chow, J. W. , and D. S. Stokic . 2011. “Force Control of Quadriceps Muscle Is Bilaterally Impaired in Subacute Stroke.” Journal of Applied Physiology 111, no. 5: 1290–1295. 10.1152/JAPPLPHYSIOL.00462.2011. 21885803

[brb370365-bib-0020] Christou, E. A. 2009. “Aging and Neuromuscular Adaptations With Practice.” In Advances in Neuromuscular Physiology of Motor Skills and Muscle Fatigue, edited by M. Shinohara , 65–79. Research Signpost.

[brb370365-bib-0021] Christou, E. A. 2011. “Aging and Variability of Voluntary Contractions.” Exercise and Sport Sciences Reviews 39, no. 2: 77–84. 10.1097/JES.0b013e31820b85ab. 21206281 PMC3631580

[brb370365-bib-0022] Christou, E. A. , and L. G. Carlton . 2001. “Old Adults Exhibit Greater Motor Output Variability Than Young Adults Only During Rapid Discrete Isometric Contractions.” Journals of Gerontology—Series A Biological Sciences and Medical Sciences 56, no. 12: B524–B532. 10.1093/gerona/56.12.B524. 11723145

[brb370365-bib-0023] Christou, E. A. , and L. G. Carlton . 2002. “Age and Contraction Type Influence Motor Output Variability in Rapid Discrete Tasks.” Journal of Applied Physiology 93, no. 2: 489–498. 10.1152/japplphysiol.00335.2001. 12133855

[brb370365-bib-0024] Christou, E. A. , B. Poston , J. A. Enoka , and R. M. Enoka . 2007. “Different Neural Adjustments Improve Endpoint Accuracy with Practice in Young and Old Adults.” Journal of Neurophysiology 97, no. 5: 3340–3350. 10.1152/jn.01138.2006. 17376846

[brb370365-bib-0025] Chung, H. J. , L. L. Weyandt , and A. Swentosky . 2014. “The Physiology of Executive functioning.” In Handbook of Executive Functioning, edited by S. Goldstein and J. A. Naglieri , 13–27. Springer Science + Business Media. 10.1007/978-1-4614-8106-5_2.

[brb370365-bib-0026] Cirstea, M. C. , and M. F. Levin . 2000. “Compensatory Strategies for Reaching in Stroke.” Brain 123, no. 5: 940–953. 10.1093/BRAIN/123.5.940. 10775539

[brb370365-bib-0027] Cohen, J. 2013. Statistical Power Analysis for the Behavioral Sciences. Routledge. 10.4324/9780203771587.

[brb370365-bib-0028] Danieli, K. , A. Guyon , and I. Bethus . 2023. “Episodic Memory Formation: A Review of Complex Hippocampus Input Pathways.” Progress in Neuro‐Psychopharmacology and Biological Psychiatry 126: 110757. 10.1016/J.PNPBP.2023.110757. 37086812

[brb370365-bib-0029] Delmas, S. , Y. J. Choi , M. Komer , M. Weintraub , B. Yacoubi , and E. A. Christou . 2021. “Older Adults Use a Motor Plan That Is Detrimental to Endpoint Control.” Scientific Reports 11, no. 1: 7562. 10.1038/s41598-021-86959-9. 33828133 PMC8027829

[brb370365-bib-0030] Desmurget, M. , and S. Grafton . 2000. “Forward Modeling Allows Feedback Control for Fast Reaching Movements.” Trends in Cognitive Sciences 4, no. 11: 423–431. 10.1016/S1364-6613(00)01537-0. 11058820

[brb370365-bib-0031] Diamond, A. 2013. “Executive Functions.” Annual Review of Psychology 64, no. 1: 135–168. 10.1146/annurev-psych-113011-143750. PMC4084861 23020641

[brb370365-bib-0032] Duncan, P. W. , K. J. Sullivan , A. L. Behrman , et al. 2011. “Body‐Weight‐Supported Treadmill Rehabilitation After Stroke.” New England Journal of Medicine 364, no. 21: 2026–2036. 10.1056/NEJMOA1010790. 21612471 PMC3175688

[brb370365-bib-0033] Elliott, R. 2003. “Executive Functions and Their Disorders.” British Medical Bulletin 65: 49–59. 10.1093/BMB/65.1.49. 12697616

[brb370365-bib-0034] Eng, J. J. , C. M. Kim , and D. L. MacIntyre . 2002. “Reliability of Lower Extremity Strength Measures in Persons With Chronic Stroke.” Archives of Physical Medicine and Rehabilitation 83, no. 3: 322–328. 10.1053/APMR.2002.29622. 11887111 PMC3489912

[brb370365-bib-0035] Faria‐Fortini, I. , M. L. Basílio , J. C. Polese , et al. 2017. “Strength Deficits of the Paretic Lower Extremity Muscles Were the Impairment Variables That Best Explained Restrictions in Participation After Stroke.” Disability and Rehabilitation 39, no. 21: 2158–2163. 10.1080/09638288.2016.1219397. 27599131

[brb370365-bib-0036] Faria‐Fortini, I. , S. M. Michaelsen , J. G. Cassiano , and L. F. Teixeira‐Salmela . 2011. “Upper Extremity Function in Stroke Subjects: Relationships Between the International Classification of Functioning, Disability, and Health Domains.” Journal of Hand Therapy 24, no. 3: 257–265. 10.1016/J.JHT.2011.01.002. 21420279

[brb370365-bib-0037] Fjell, A. M. , L. T. Westlye , I. K. Amlien , and K. B. Walhovd . 2011. “Reduced White Matter Integrity Is Related to Cognitive Instability.” Journal of Neuroscience 31, no. 49: 18060–18072. 10.1523/JNEUROSCI.4735-11.2011. 22159119 PMC6634144

[brb370365-bib-0038] Ghez, C. , and J. Gordon . 1987. “Trajectory Control in Targeted Force Impulses—I. Role of Opposing Muscles.” Experimental Brain Research 67, no. 2: 225–240. 10.1007/BF00248545. 3622686

[brb370365-bib-0039] Gordon, J. , and C. Ghez . 1987. “Trajectory Control in Targeted Force Impulses III. Compensatory Adjustments for Initial Errors.” Experimental Brain Research 67: 253–269. 10.1007/BF00248547. 3622688

[brb370365-bib-0040] Gordon, J. , M. F. Ghilardi , and C. Ghez . 1994. “Accuracy of Planar Reaching Movements—I. Independence of Direction and Extent Variability.” Experimental Brain Research 99, no. 1: 97–111. 10.1007/BF00241415. 7925800

[brb370365-bib-0041] Hachinski, V. , C. Iadecola , R. C. Petersen , et al. 2006. “National Institute of Neurological Disorders and Stroke–Canadian Stroke Network Vascular Cognitive Impairment Harmonization Standards.” Stroke 37, no. 9: 2220–2241. 10.1161/01.STR.0000237236.88823.47. 16917086

[brb370365-bib-0042] Hammerbeck, U. , N. Yousif , D. Hoad , R. Greenwood , J. Diedrichsen , and J. C. Rothwell . 2017. “Chronic Stroke Survivors Improve Reaching Accuracy by Reducing Movement Variability at the Trained Movement Speed.” Neurorehabilitation and Neural Repair 31, no. 6: 499–508. 10.1177/1545968317693112. 28506150

[brb370365-bib-0043] Harrison, J. K. , K. S. McArthur , and T. J. Quinn . 2013. “Assessment Scales in Stroke: Clinimetric and Clinical Considerations.” Clinical Interventions in Aging 8: 201–211. 10.2147/CIA.S32405. 23440256 PMC3578502

[brb370365-bib-0044] Hayes, S. , C. Donnellan , and E. Stokes . 2013. “Associations Between Executive Function and Physical Function Poststroke: A Pilot Study.” Physiotherapy 99, no. 2: 165–171. 10.1016/J.PHYSIO.2012.05.002. 23219633

[brb370365-bib-0045] Haynes, B. I. , S. Bauermeister , and D. Bunce . 2017. “A Systematic Review of Longitudinal Associations Between Reaction Time Intraindividual Variability and Age‐Related Cognitive Decline or Impairment, Dementia, and Mortality.” Journal of the International Neuropsychological Society 23: 431–445. 10.1017/S1355617717000236. 28462758

[brb370365-bib-0046] Hornby, T. G. , C. E. Henderson , A. Plawecki , et al. 2019. “Contributions of Stepping Intensity and Variability to Mobility in Individuals Poststroke.” Stroke 50, no. 9: 2492–2499. 10.1161/STROKEAHA.119.026254. 31434543 PMC7241260

[brb370365-bib-0047] Hu, X. , and K. M. Newell . 2011. “Motor‐Unit Pool Model of Continuous and Discrete Force Variability.” Motor Control 15, no. 4: 439–455. 10.1123/mcj.15.4.439. 21941020

[brb370365-bib-0048] Hultsch, D. F. , and S. W. S. MacDonald . 2004. “Intraindividual Variability in Performance as a Theoretical Window Onto Cognitive Aging.” In New Frontiers in Cognitive Aging, edited by R. Dixon , L. Backman , and L.‐G. Nilsson , 65–88. Oxford University Press.

[brb370365-bib-0049] Hultsch, D. F. , S. W. S. MacDonald , and R. A. Dixon . 2002. “Variability in Reaction Time Performance of Younger and Older Adults.” Journals of Gerontology: Series B 57, no. 2: P101–P115. 10.1093/GERONB/57.2.P101. 11867658

[brb370365-bib-0050] Hultsch, D. F. , S. W. S. MacDonald , M. A. Hunter , J. Levy‐Bencheton , and E. Strauss . 2000. “Intraindividual Variability in Cognitive Performance in Older Adults: Comparison of Adults With Mild Dementia, Adults With Arthritis, and Healthy Adults.” Neuropsychology 14, no. 4: 588–598. 10.1037//0894-4105.14.4.588. 11055261

[brb370365-bib-0051] Hyndman, D. , and A. Ashburn . 2003. “People With Stroke Living in the Community: Attention Deficits, Balance, ADL Ability and Falls.” Disability and Rehabilitation 25, no. 15: 817–822. 10.1080/0963828031000122221. 12851091

[brb370365-bib-0052] Hyndman, D. , R. M. Pickering , and A. Ashburn . 2008. “The Influence of Attention Deficits on Functional Recovery post Stroke During the First 12 Months After Discharge From Hospital.” Journal of Neurology, Neurosurgery and Psychiatry 79: 656–663. 10.1136/jnnp.2007.125609. 17872979

[brb370365-bib-0053] Jackson, J. D. , D. A. Balota , J. M. Duchek , and D. Head . 2012. “White Matter Integrity and Reaction Time Intraindividual Variability in Healthy Aging and Early‐stage Alzheimer Disease.” Neuropsychologia 50, no. 3: 357–366. 10.1016/J.NEUROPSYCHOLOGIA.2011.11.024. 22172547 PMC3302689

[brb370365-bib-0054] Jaeger, J. 2018. “Digit Symbol Substitution Test.” Journal of Clinical Psychopharmacology 38, no. 5: 513–519. 10.1097/JCP.0000000000000941. 30124583 PMC6291255

[brb370365-bib-0055] Jurica, P. J. , C. L. Leitten , and S. Mattis . 2001. Dementia Rating Scale‐2: DRS‐2: Professional Manual. Psychological Assessment Resources.

[brb370365-bib-0056] Kawahira, K. , M. Shimodozono , A. Ogata , et al. 2005. “Impaired Visuo‐Motor Skills in the Unaffected Lower Limb of Patients With Stroke.” International Journal of Neuroscience 115, no. 9: 1315–1332. 10.1080/00207450590934561. 16048808

[brb370365-bib-0057] Kim, S. , L. G. Carlton , Y. T. Liu , and K. M. Newell . 1999. “Impulse and Movement Space—Time Variability.” Journal of Motor Behavior 31, no. 4: 341–357. 10.1080/00222899909600999. 11177642

[brb370365-bib-0058] Lindberg, P. G. , N. Roche , J. Robertson , A. Roby‐Brami , B. Bussel , and M. A. Maier . 2012. “Affected and Unaffected Quantitative Aspects of Grip Force Control in Hemiparetic Patients After Stroke.” Brain Research 1452: 96–107. 10.1016/J.BRAINRES.2012.03.007. 22464180

[brb370365-bib-0059] Liu, J. , Q. Wang , F. Liu , et al. 2017. “Altered Functional Connectivity in Patients With Post‐Stroke Memory Impairment: A Resting fMRI Study.” Experimental and Therapeutic Medicine 14, no. 3: 1919–1928. 10.3892/ETM.2017.4751. 28962104 PMC5609161

[brb370365-bib-0060] Liu‐Ambrose, T. , M. Y. C. Pang , and J. J. Eng . 2007. “Executive Function Is Independently Associated With Performances of Balance and Mobility in Community‐Dwelling Older Adults After Mild Stroke: Implications for Falls Prevention.” Cerebrovascular Diseases 23, no. 2–3: 203–210. 10.1159/000097642. 17143004 PMC4492718

[brb370365-bib-0061] Lodha, N. , S. A. Coombes , and J. H. Cauraugh . 2012. “Bimanual Isometric Force Control: Asymmetry and Coordination Evidence Post Stroke.” Clinical Neurophysiology 123, no. 4: 787–795. 10.1016/J.CLINPH.2011.08.014. 21924949

[brb370365-bib-0062] Lodha, N. , H. Moon , C. Kim , T. Onushko , E. A. Christou , and S. Kritchevsky . 2016. “Motor Output Variability Impairs Driving Ability in Older Adults.” Journals of Gerontology. Series A, Biological Sciences and Medical Sciences 71, no. 12: glw013. 10.1093/gerona/glw013. 26935111

[brb370365-bib-0063] Lodha, N. , S. K. Naik , S. A. Coombes , and J. H. Cauraugh . 2010. “Force Control and Degree of Motor Impairments in Chronic Stroke.” Clinical Neurophysiology 121, no. 11: 1952–1961. 10.1016/J.CLINPH.2010.04.005. 20435515

[brb370365-bib-0064] Lodha, N. , P. Patel , A. Casamento‐Moran , K. Gauger , and E. A. Christou . 2019. “Endpoint Accuracy of Goal‐Directed Ankle Movements Correlates to Over‐Ground Walking in Stroke.” Clinical Neurophysiology 130, no. 6: 1008–1016. 10.1016/J.CLINPH.2019.03.030. 31005051

[brb370365-bib-0065] Lodha, N. , P. Patel , A. Casamento‐Moran , E. Hays , S. N. Poisson , and E. A. Christou . 2019. “Strength or Motor Control: What Matters in High‐Functioning Stroke?” Frontiers in Neurology 9: 1160. 10.3389/FNEUR.2018.01160. 30687217 PMC6333669

[brb370365-bib-0066] Lopez, F. V. , L. E. Kenney , A. Ratajska , C. E. Jacobson , and D. Bowers . 2021. “What Does the Dementia Rating Scale‐2 Measure? The Relationship of Neuropsychological Measures to DRS‐2 Total and Subscale Scores in Non‐Demented Individuals With Parkinson's Disease.” Clinical Neuropsychologist 37, no. 1: 174. 10.1080/13854046.2021.1999505. 34779350 PMC9107526

[brb370365-bib-0067] Lövdén, M. , L. Bäckman , U. Lindenberger , S. Schaefer , and F. Schmiedek . 2010. “A Theoretical Framework for the Study of Adult Cognitive Plasticity.” Psychological Bulletin 136, no. 4: 659–676. 10.1037/A0020080. 20565172

[brb370365-bib-0068] MacDonald, S. W. S. , L. Nyberg , and L. Bäckman . 2006. “Intra‐Individual Variability in Behavior: Links to Brain Structure, Neurotransmission and Neuronal Activity.” Trends in Neurosciences 29, no. 8: 474–480. 10.1016/J.TINS.2006.06.011. 16820224

[brb370365-bib-0069] Miyake, A. , N. P. Friedman , M. J. Emerson , A. H. Witzki , A. Howerter , and T. D. Wager . 2000. “The Unity and Diversity of Executive Functions and Their Contributions to Complex ‘Frontal Lobe’ Tasks: A Latent Variable Analysis.” Cognitive Psychology 41, no. 1: 49–100. 10.1006/cogp.1999.0734. 10945922

[brb370365-bib-0070] Mukherjee, M. , P. Koutakis , K. C. Siu , P. B. Fayad , and N. Stergiou . 2013. “Stroke Survivors Control the Temporal Structure of Variability During Reaching in Dynamic Environments.” Annals of Biomedical Engineering 41, no. 2: 366–376. 10.1007/S10439-012-0670-9. 23064865

[brb370365-bib-0071] Nys, G. , E. De Haan , G. M. S. Nys , et al. 2007. “Cognitive Disorders in Acute Stroke: Prevalence and Clinical Determinants.” Cerebrovascular Diseases 23: 408–416. 10.1159/000101464. 17406110

[brb370365-bib-0072] Ojala‐Oksala, J. , H. Jokinen , V. Kopsi , et al. 2012. “Educational History Is an Independent Predictor of Cognitive Deficits and Long‐Term Survival in Postacute Patients With Mild to Moderate Ischemic Stroke.” Stroke 43, no. 11: 2931–2935. 10.1161/STROKEAHA.112.667618. 22935400

[brb370365-bib-0073] Patel, P. , S. R. Kaingade , A. Wilcox , and N. Lodha . 2020. “Force Control Predicts Fine Motor Dexterity in High‐Functioning Stroke Survivors.” Neuroscience Letters 729: 135015. 10.1016/J.NEULET.2020.135015. 32360934

[brb370365-bib-0074] Patel, P. , and N. Lodha . 2019. “Dynamic Bimanual Force Control in Chronic Stroke: Contribution of Non‐Paretic and Paretic Hands.” Experimental Brain Research 237, no. 8: 2123–2133. 10.1007/S00221-019-05580-5. 31197412

[brb370365-bib-0075] Reitan, R. M. 1992. Trail Making Test: Manual for Administration and Scoring: Reitan Neuropsychology Laboratory. Reitan Neuropsychology Laboratory.

[brb370365-bib-0076] Rohrer, B. , S. Fasoli , H. I. Krebs , et al. 2002. “Movement Smoothness Changes During Stroke Recovery.” Journal of Neuroscience 22, no. 18: 8297–8304. 10.1523/JNEUROSCI.22-18-08297.2002. 12223584 PMC6758113

[brb370365-bib-0077] Rosenich, E. , B. Hordacre , C. Paquet , S. A. Koblar , and S. L. Hillier . 2020. “Cognitive Reserve as an Emerging Concept in Stroke Recovery.” Neurorehabilitation and Neural Repair 34, no. 3: 187–199. 10.1177/1545968320907071. 32089097

[brb370365-bib-0078] Seidler, R. D. , D. C. Noll , and G. Thiers . 2004. “Feedforward and Feedback Processes in Motor Control.” NeuroImage 22, no. 4: 1775–1783. 10.1016/j.neuroimage.2004.05.003. 15275933

[brb370365-bib-0079] Shadmehr, R. , M. A. Smith , and J. W. Krakauer . 2010. “Error Correction, Sensory Prediction, and Adaptation in Motor Control.” Annual Review of Neuroscience 33: 89–108. 10.1146/annurev-neuro-060909-153135. 20367317

[brb370365-bib-0080] Shmuelof, L. , J. W. Krakauer , and P. Mazzoni . 2012. “How Is a Motor Skill Learned? Change and Invariance at the Levels of Task Success and Trajectory Control.” Journal of Neurophysiology 108, no. 2: 578–594. 10.1152/JN.00856.2011. 22514286 PMC3404800

[brb370365-bib-0081] Sofia Costa, A. , I. Dogan , J. B. Schulz , and K. Reetz . 2019. “The Clinical Neuropsychologist Going Beyond the Mean: Intraindividual Variability of Cognitive Performance in Prodromal and Early Neurodegenerative Disorders.” Clinical Neuropsychologist 33, no. 2: 369–389. 10.1080/13854046.2018.1533587. 30663511

[brb370365-bib-0082] Stapleton, T. , A. Ashburn , and E. Stack . 2001. “A Pilot Study of Attention Deficits, Balance Control and Falls in the Subacute Stage Following Stroke.” Clinical Rehabilitation 15, no. 4: 437–444. 10.1191/026921501678310243. 11518445

[brb370365-bib-0083] Stiles, J. , N. A. Akshoomoff , and F. Haist . 2020. “The Development of Visuospatial Processing.” Neural Circuit and Cognitive Development 359–393. 10.1016/B978-0-12-814411-4.00017-2.

[brb370365-bib-0084] Stock, R. , G. Thrane , T. Askim , A. Anke , and P. J. Mork . 2019. “Development of Grip Strength During the First Year After Stroke.” Journal of Rehabilitation Medicine 51, no. 4: 248–256. 10.2340/16501977-2530. 30848829

[brb370365-bib-0085] Stuss, D. T. , J. Pogue , L. Buckle , and J. Bondar . 1994. “Characterization of Stability of Performance in Patients with Traumatic Brain Injury: Variability and Consistency on Reaction Time Tests.” Neuropsychology 8, no. 3: 316–324. 10.1037/0894-4105.8.3.316.

[brb370365-bib-0086] Sunderland, A. , M. P. Bowers , S.‐M. Sluman , D. J. Wilcock , and M. E. Ardron . 1999. “Impaired Dexterity of the Ipsilateral Hand after Stroke and the Relationship to Cognitive Deficit.” Stroke 30, no. 5: 949–955. 10229726 10.1161/01.str.30.5.949

[brb370365-bib-0087] Teasell, R. W. , N. C. Foley , S. K. Bhogal , and M. R. Speechley . 2015. “An Evidence‐Based Review of Stroke Rehabilitation.” Topics in Stroke Rehabilitation 10, no. 1: 29–58. 10.1310/8YNA-1YHK-YMHB-XTE1. 12970830

[brb370365-bib-0088] van Beers, R. J. 2009. “Motor Learning Is Optimally Tuned to the Properties of Motor Noise.” Neuron 63, no. 3: 406–417. 10.1016/j.neuron.2009.06.025. 19679079

[brb370365-bib-0089] Van Vugt, F. T. , and D. J. Ostry . 2018. “The Structure and Acquisition of Sensorimotor Maps.” Journal of Cognitive Neuroscience 30, no. 3: 290–306. 10.1162/JOCN_A_01204. 29131742

[brb370365-bib-0090] Weyandt, L. L. 2009. “Executive Functions and Attention Deficit Hyperactivity Disorder.” ADHD Report 17, no. 6: 1–7. 10.1521/adhd.2009.17.6.1.

[brb370365-bib-0091] Yacoubi, B. , and E. A. Christou . 2024. “Motor Output Variability in Movement Disorders: Insights From Essential Tremor.” Exercise and Sport Sciences Reviews 52, no. 3: 95–101. 10.1249/JES.0000000000000338. 38445865

[brb370365-bib-0092] Zelazo, P. D. , and U. Müller . 2010. “Executive Function in Typical and Atypical Development.” In The Wiley‐Blackwell Handbook of Childhood Cognitive Development, edited by U. Goswami , 574–603. Wiley Blackwell. 10.1002/9781444325485.ch22.

